# Molecular Insights into *RrMYB5* Promote Flavonoid Accumulation in *Rosa roxburghii*

**DOI:** 10.3390/plants15162428

**Published:** 2026-08-09

**Authors:** Linfang Zhang, Linlu Si, Mao Wu, Xiaolong Huang, Huiqing Yan

**Affiliations:** 1 School of Life Sciences, Guizhou Normal University, Guiyang 550001, China; zhanglinfang@gznu.edu.cn (L.Z.); silinlu211@gznu.edu.cn (L.S.); 232100100410@gznu.edu.cn (M.W.); 2 Key Laboratory of Plant Physiology and Development Regulation, Guizhou Normal University, Guiyang 550001, China; 3 Key Laboratory of State Forestry Administration on Biodiversity Conservation in Mountainous Karst Area of Southwestern China, Guizhou Normal University, Guiyang 550001, China

**Keywords:** *Rosa roxburghii*, flavonoids, MYB, DAP-seq

## Abstract

*Rosa roxburghii* Tratt is characterized by its abundant flavonoid content. However, the mechanisms underlying MYB-mediated regulation of flavonoid biosynthesis in *R. roxburghii* remain largely unknown. In this study, we found that flavonoid accumulation was markedly higher in fruits. By WGCNA of differentially expressed genes (DEGs) with flavonoid accumulation profiles, we identified *RrMYB5* as a key regulatory factor in flavonoid biosynthesis of *R. roxburghii*. RrMYB5 contained characteristic R2R3 domains and a conserved PA1-type motif YEEYLQALL. It was localized exclusively to the nucleus. The qRT-PCR analysis showed that *RrMYB5* was constitutively expressed, with peak expression occurring at the rapid fruit expansion stage. The total soluble flavonoid accumulation in *R. roxburghii* calli was substantially increased by overexpression of *RrMYB5*. Further LC–MS-based metabolomic analysis revealed significant enrichment of flavonols and proanthocyanidins in *RrMYB5*-OE calli. Consistently, the transcript levels of *RrLAR* (*Rr404249*) and *RrANR* (*Rr300417*) were markedly elevated in *RrMYB5*-overexpressing calli. Moreover, DAP-seq analysis suggested that RrMYB5 might directly bind the promoters of flavonoid structural genes. Subsequent yeast one-hybrid and dual-luciferase assays confirmed that *RrFLS* (*Rr101307*) and *RrF3H* (*Rr306546*) were direct downstream targets of RrMYB5. These findings indicated that RrMYB5 promoted the expression of flavonol and flavanol biosynthetic genes through different regulatory routes. Thus, our study elucidates the regulatory role of *RrMYB5* in flavonoid biosynthesis and provides a valuable molecular target for improving the quality and utilization of *R. roxburghii*.

## 1. Introduction

Flavonoids sharing a conserved carbon scaffold (C6-C3-C6) constitute the largest class of phenolic compounds and are divided into several major groups, such as flavones, isoflavones, dihydroflavonols, flavonols, anthocyanins, proanthocyanidins (PAs) and so on [1]. In plants, flavonoid biosynthesis originates from phenylalanine and proceeds by a coordinated cascade of enzymes, beginning with phenylalanine ammonia-lyase (PAL), followed by cinnamate 4-hydroxylase (C4H), and 4-coumarate-CoA ligase (4CL). Then chalcone synthase (CHS) catalyzes the condensation of 4-coumaroyl-CoA with three molecules of malonyl-CoA to convert chalcone by chalcone isomerase (CHI) into flavanone. Flavanone is further hydroxylated by flavanone 3-hydroxylase (F3H), flavonoid 3′,5′-hydroxylase (F3′5′H) to produce dihydroflavonols [2]. Subsequently, flavonol synthesis (FLS) catalyzes dihydroflavonols to yield flavonols, such as kaempferol and quercetin. In a parallel branch, dihydroflavonol 4-reductase (DFR) catalyzes the formation of leucoanthocyanidins, which are further converted by anthocyanidin synthase (ANS) and flavonoid 3-O-glucosyltransferase (UGT) into anthocyanins sequestered in vacuoles [3]. In addition, the PA-specific branch is mediated by leucoanthocyanidin reductase (LAR) and anthocyanidin reductase (ANR), which directly generate the flavan-3-ol unit [4]. Polymers composed of diverse flavan-3-ol monomers, including catechin, epicatechin, and epigallocatechin, are especially abundant in tannin-rich plants and play important physiological roles [5]. 

The expressions of structural genes in flavonoid biosynthesis are meticulously orchestrated by transcription factors (TFs), especially MYBs [5]. These proteins are composed of one or four R-repeated domains and classified into four subclasses: 1R-MYB, R2R3-MYB, R1R2R3-MYB, and 4R-MY [6,7]. R2R3-MYBs comprise two conserved N-terminal domains and a defined highly variable C-terminal region that generally harbors an activation or repression domain, thereby conferring regulatory specificity in flavonoid metabolism [8]. The N-terminal domain mediates physical interaction with basic helix–loop–helix (bHLH) transcription factors, serving as a basis for the assembly of the MYB–bHLH–WD40 (MBW) complex to synergistically regulate flavonoid biosynthesis. In *Arabidopsis*, AtMYB75 (PAP1), AtMYB90 (PAP2), AtMYB113, and AtMYB114 have been shown to strongly promote anthocyanin biosynthesis by activating flavonoid structural genes [9,10]. Flavanol and flavones are predominantly most active in the foliage of *Spuriopimpinella brachycarpa* under the rigorous regulatory control of MYB-mediated regulatory control [11]. In addition, the genome of *Camellia sinensis* harbors 10 PA-related, one anthocyanin-related, and three flavonol-related MYBs. Additionally, PA1-type MYBs, such as *Vitis vinifera* MYB5b and *Rosa rugosa* MYB5, are generally considered activators of PA biosynthesis, activating the transcripts of *DFR*, *LAR*, and *ANR* [12,13]. Nevertheless, the effects of PA1-type MYBs on anthocyanin accumulation varied among different species. For instance, ectopic overexpression of *CsMYB5a* inhibited anthocyanin accumulation in tobacco, whereas overexpression of *CsMYB5e* did not affect anthocyanin levels [14]. Thus, the species functions of PA1-type MYBs in regulating flavonoid biosynthetic structural genes should be further elucidated.

*Rosa roxburghii* is a perennial deciduous clumping shrub of the Rosaceae family and is distinguished for its rich repertoire of bioactive compounds, such as ascorbic acid and polyphenols, which confer potent strong antioxidant and antimicrobial activities and their protective functions against various stresses [15]. The pronounced antioxidant capacity of *R. roxburghii* is largely attributable to its high abundance of flavonoids, particularly flavonols, including quercetin and its derivatives, as well as proanthocyanidins (PAs), such as catechins and their derivative [16]. Some studies on the fruits of *R. roxburghii* proved that they could protect against chronic inflammation, aging and organic atrophy due to its bioactive flavonoid [17]. Moreover, the fruit is often used as a raw material for Chinese medicinal extraction in the food industry [18]. Thus, studying the potential molecular mechanism of flavonoid biosynthesis is of great importance to explore the hidden potential of *R. roxburghii* fruit. Although genome-wide investigations of transcription factors in *R. roxburghii* have been reported in recent years, these studies have primarily focused on regulatory networks associated with vitamin C biosynthesis and other fruit-related traits [19]. By contrast, the roles of MYB transcription factors in regulating structural genes involved in flavonoid biosynthesis remain largely unexplored.

In this study, we firstly determined a key regulator, *RrMYB5,* in regulating flavonoid accumulation. After we analyzed its structure and subcellular location, its spatiotemporal transcript profiles were determined by qRT-PCR. Through overexpression of *RrMYB5* in *R. roxburghii* calli, flavonoid accumulation was determined by LC-MS analysis. Further DAP-seq analysis, as well as yeast one-hybrid and luciferase reporter assays, further confirmed the flavonoid structural genes were directly regulated by RrMYB5. Thus, these findings provide new insight into the regulatory networks governing flavonoid biosynthesis in *R. roxburghii* and offer a promising molecular target for the processing and utilization of functional compounds from *R. roxburghii* products.

## 2. Results

### 2.1. Flavonoid Content and Co-Expression Modules in Various Organs of R. roxburghii

**Figure 1:**
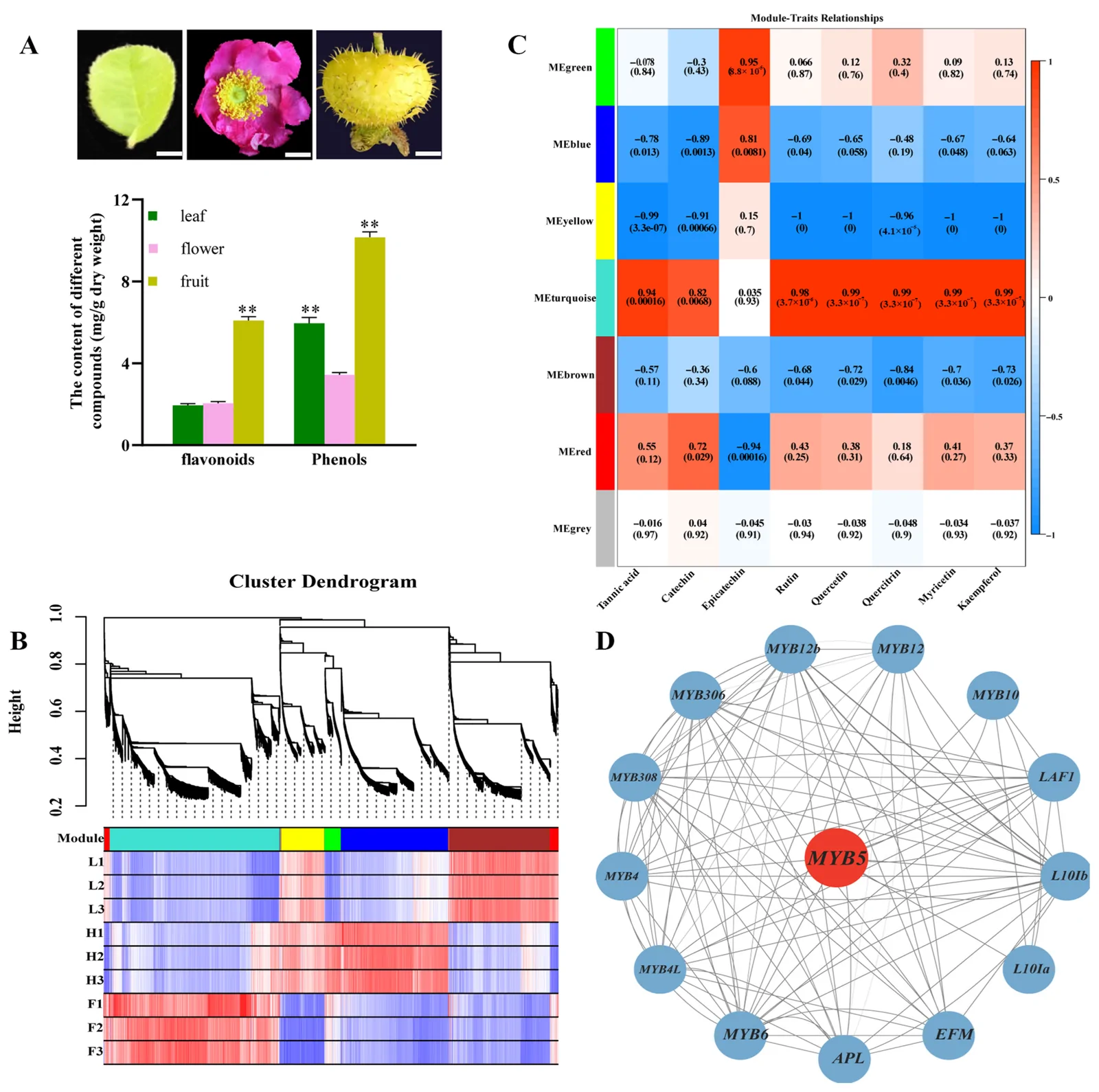
*RrMYB5* might play a critical role in regulating flavonoid accumulation. (**A**) Contents of total phenols and soluble flavonoids in different tissues of *R. roxburghii*. Values are expressed as mg/g of the dry weight. ** indicates significant difference at *p* ≤ 0.01, determined by Student’s *t*-test. Scale bar = 1 cm. (**B**) Hierarchical clustering dendrogram based on consensus topological overlap dissimilarity. The horizontal color bar denotes the corresponding module assignments, with each color representing a distinct module comprising a cluster of highly interconnected genes. The heatmap below displays module expression profiles across leaves, flowers, and fruits of *R. roxburghii*. Different color indicated different clusters of DEGs. (**C**) Heatmap showing module–trait relationships between consensus module eigengenes and phenolic compounds. The color coding is according to the color legend, with red indicating positive correlations and blue indicating negative correlations. (**D**) Cytoscape visualization of *RrMYBs* in MEturquoise with correlation coefficients greater than 0.8 and *p*-value less than 0.05.

Although phenolic compounds were broadly distributed across *R. roxburghii* tissues, their tissue-specific accumulation, particularly that of flavonoids, varied markedly among leaves, flowers, and fruits (Figure 1A). Fruits exhibited exceptionally high levels of both total phenolics and flavonoids, reaching 10.17 mg/g and 6.10 mg/g of dry weight, respectively, with flavonoids accounting for 59.96% of total phenolics. Although flavonoid contents were comparable in flowers and leaves, at 2.05 mg/g and 1.95 mg/g dry weight, respectively, flavonoids represented 59.59% of total phenolics in flowers but only 32.72% in leaves, suggesting that flavonoids predominated in flowers, whereas phenolic acids were more abundant in leaves. These results demonstrate that flavonoids are differentially distributed among leaves, flowers, and fruits, with maximal accumulation occurring in fruits. Therefore, identifying the key regulatory genes governing flavonoid accumulation in fruits is essential. 

To elucidate the regulatory relationships and identify pivotal genes governing flavonoid biosynthesis in fruits of *R. roxburghii*, WGCNA was conducted utilizing 21,508 non-redundant putative differentially expressed genes (DEGs) identified among three tissues. The hierarchical dendrogram displayed seven distinct co-expression modules, visualizing clusters of highly correlated transcripts as distinct branches, with each leaf representing an individual unigene and modules denoted by color bars, such as turquoise, blue, and brown, applied with a minimum module size of 50 and a merge cut height of 0.25 (Figure 1B). The heatmap below illustrates the expression profiles of these modules across leaf (L1–L3), flower (H1–H3), and fruit (F1–F3) samples, revealing sample-specific expression trends. Subsequently, the accumulations of different phenols, including catechin, epicatechin, rutin, quercetin, myricetin, and kaempferol, are displayed in Figure S1 and incorporated as phenotypic trait data to facilitate module–trait correlation analysis [16]. Notably, the MEturquoise module, comprising 5480 genes, exhibited a profound correlation with quercetin (r = 0.99, *p* = 3.3 × 10^−7^) and catechin (r = 0.82, *p* = 0.0068) (Figure 1C). To determine the critical MYBs most closely associated with flavonoid accumulation, the resulting network revealed a highly interconnected cluster centered on *MYB5* (*Rr703541*), together with *MYB4L* (*Rr300495*), *MYB306* (*Rr501773*), *MYB308* (*Rr500178*), and *L10Ib* (Rr704249), suggesting a key role for *RrMYB5* in flavonoid accumulation (Figure 1D).

### 2.2. Homology of RrMYB5 and Subcellular Location

**Figure 2:**
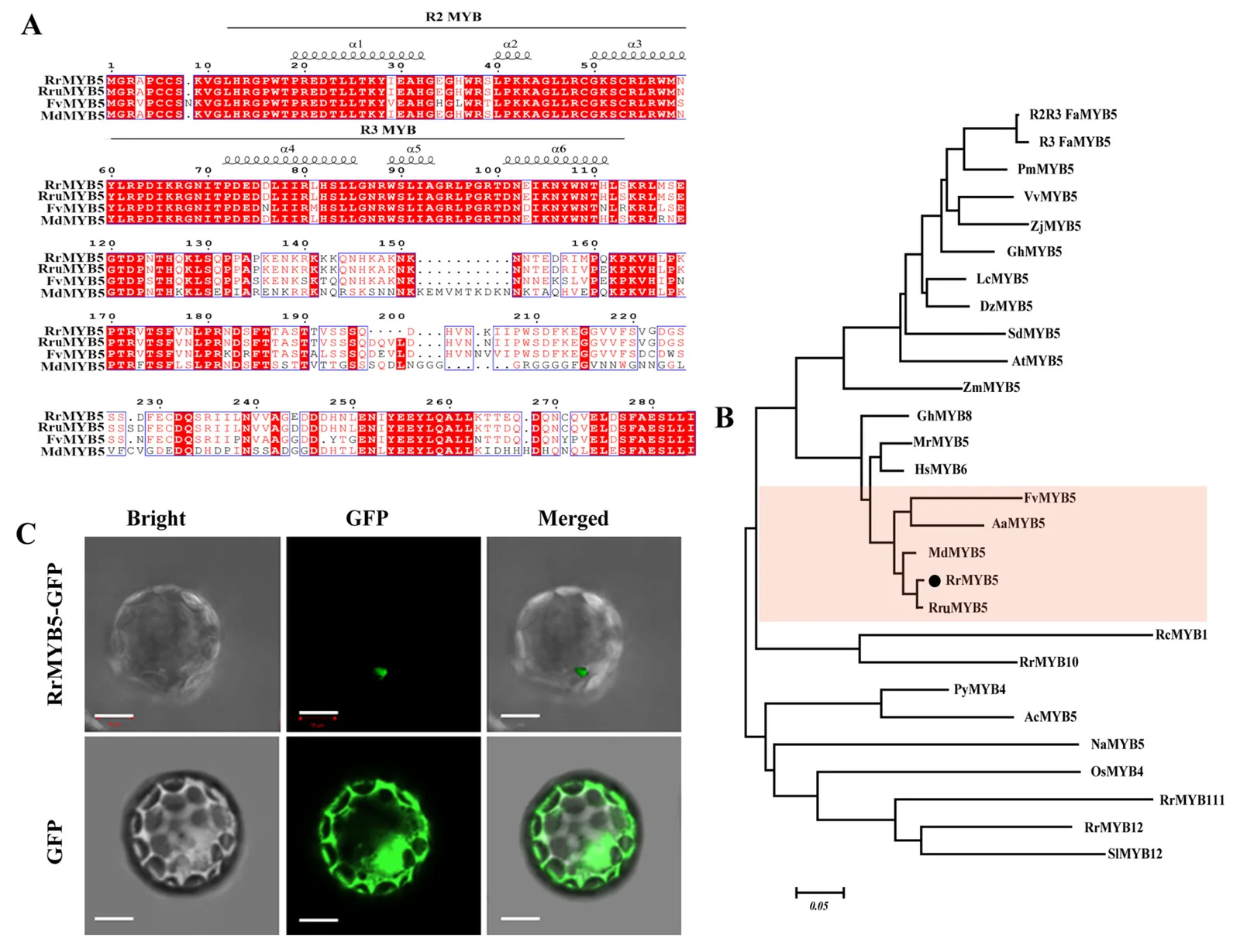
Sequence alignment, phylogenetic, and subcellular localization of RrMYB5. (**A**) Deduced amino acid sequence alignment of RrMYB5 with MYB5 homologs from *Rosa rugosa*, *Fragaria vesca*, and apple. Identical amino acids are highlighted in red, and the black line above the alignment indicates the MYB domain. The red shadow denotes the same amino acids among four species, and the blue frame indicates the highly conserved amino acids. (**B**) Phylogenetic tree of MYB5 homologs from *R. roxburghii* and others. The tree was constructed using the neighbor-joining method in MEGA7 with 1000 bootstrap replicates. GenBank accession numbers: RruMYB5 (*Rosa rugosa*), AYP10274.1; MdMYB5 (*Malus domestica*), NP_001315731; FvMYB5 (*Fragaria vesca*), XP_004301157.1; AaMYB5 (*Argentina anserina*), XP_050385281.1; AtMYB5 (*Arabidopsis thaliana*), NP_187963; HsMYB6 (*Hibiscus syriacus*), KAE8700978.1; MrMYB5 (*Morella rubra*), KAB1219664.1; GhMYB8 (*Gossypium hirsutum*), XP_016729808.1; R3-FaMYB5 (*Fragaria* × *ananassa*), AFL02459; R2R3-FaMYB5, MW700311; RcMYB1 (*Rosa chinensis*), XP_024189921.1; VvMYB5 (*Vitis vinifera*), NP_001267854.1; OsMYB4 (*Oryza sativa*), Q7XBH4; LcMYB5 (*Litchi chinensis*), QRV61382.1; ZmMYB5 (*Zea mays*), XP_008656780; SdMYB5 (*Salvia divinorum*), KAL1557441.1; NaMYB5 (*Nicotiana attenuata*), OIT28820.1; AcMYB5 (*Allium cepa*), AQP25674.1; PmMYB5 (*Prunus mume*), XP_008220661; ChMYB8 (*Gossypium hirsutum*), XP_016729808.1; ZjMYB5 (*Ziziphus jujuba*), XP_015888701.3; DzMYB5 (*Durio zibethinus*), XP_022769742.1; PhMYB4 (*Petunia hybrida*), ACB12238.1; RrMYB111 (*Rosa rugosa*), XM_062138075; RrMYB1, XM_062132433; RrMYB10, CCA29099.1; SlMYB12, NP_001234401.1. The black dot indicates MYB5 from *R. roxburghi*. (**C**) Nuclear localization of RrMYB5. *Arabidopsis* protoplasts were transiently transformed with an empty vector or a 35S::*RrMYB5*-GFP. The empty vector served as the positive control. GFP, green fluorescence protein. Scale bars = 5 μm.

Given that *RrMYB5* might function as a critical positive regulator of flavonoid accumulation, detailed analysis revealed that its full-length cDNA of *RrMYB5* contained an open reading frame (ORF) of 843 bp, encoding 280 amino acids (aa). Structurally, *RrMYB5* harbors two conserved MYB-typical R repeats, with each motif consisting of approximately 50 aa residues in tandem (spanning positions 13~63 and 66~114) (Figure 2A). Sequence alignment of *RrMYB5* with MYB5 orthologs from rose (*Rosa rugosa*), woodland strawberry (*Fragaria vesca*), and apple (*Malus domestica*) revealed more than 90% similarity and the presence of a conserved C-terminal MYBPA-type motif (YEEYLQALL), suggesting a potential role in activating *LAR* and *ANR* expression in the proanthocyanidin pathway [20].

Phylogenetic analysis showed that *RrMYB5* most closely resembled homologs from rose and apple, as well as closely related sequences from *Argentina anserina* and woodland strawberry within Rosaceae, suggesting that these proteins may share conserved functions in flavonoid accumulation (Figure 2B). To determine the precise subcellular localization of RrMYB5, an RrMYB5-GFP fusion protein was transiently expressed in *Arabidopsis* protoplasts. Upon excitation with 488 nm light, protoplasts expressing the 35S::GFP exhibited ubiquitous green fluorescence throughout the entire cell, including the plasma membrane, cytoplasm, and nucleus, serving as a positive control. By contrast, the fluorescent signal in protoplasts expressing 35S::*RrMYB5*-GFP was confined exclusively to the nucleus, suggesting it is a nuclear-localized protein (Figure 2C).

### 2.3. Dynamics of RrMYB5 Expression at Different Stages of Fruit Development

**Figure 3:**
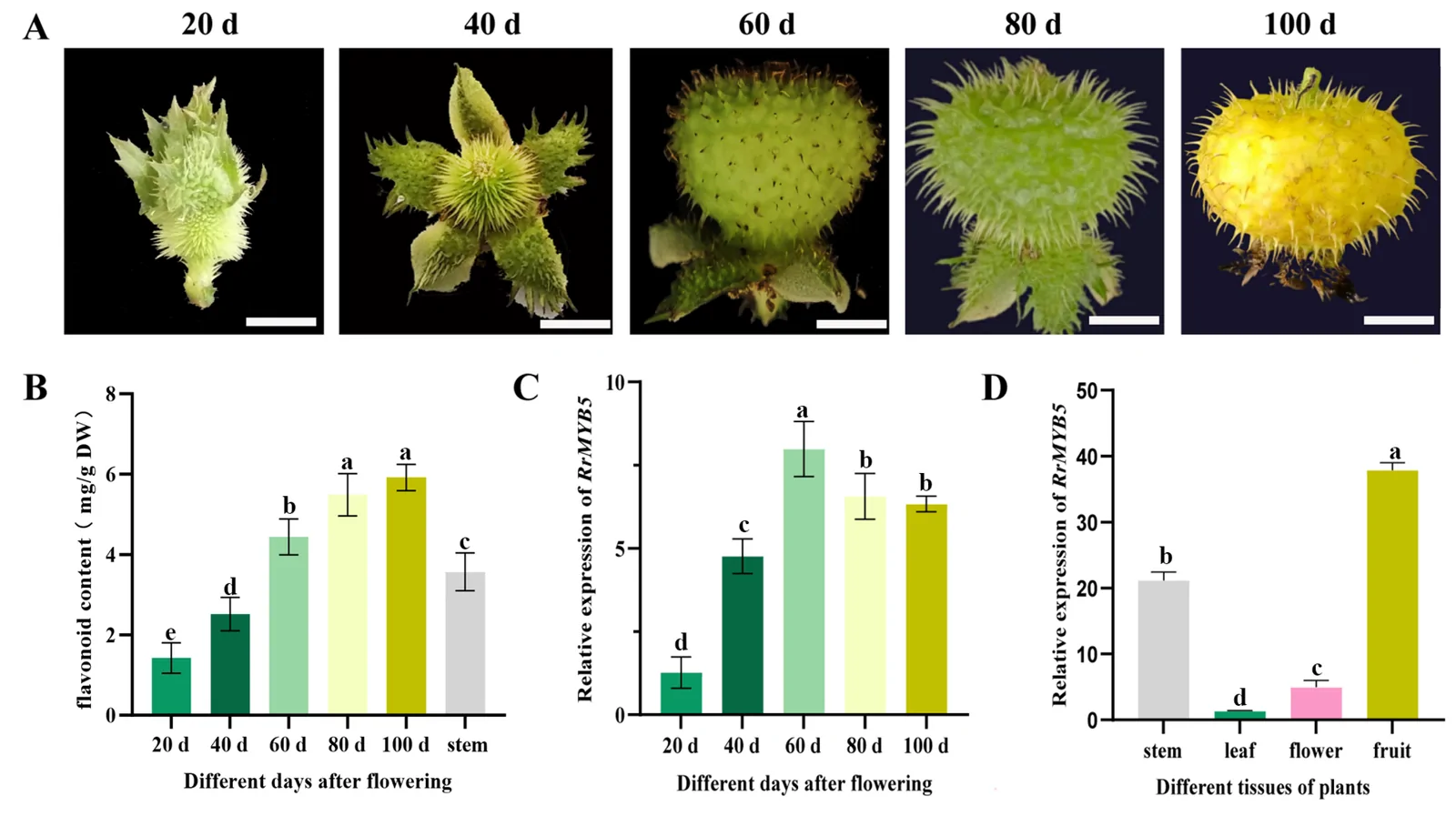
Expression profiles of *RrMYB5* across fruit developmental stages and tissues. (**A**) Phenotypes of *R. roxburghii* fruits at 20, 40, 60, 80, and 100 days after flowering (DAF). Scale bar = 1 cm. (**B**–**D**) Flavonoid contents (**B**) and relative expression level of *RrMYB5* at different fruit stages (**C**) or tissues of *R. roxburghii* (**D**). Different letters above the bars indicate significant differences at *p* ≤ 0.05.

To characterize the spatiotemporal expression profile of *RrMYB5* in *R. roxburghii*, we examined its transcript abundance in fruits at five developmental stages and across different tissues. As fruits develop from the receptacle, the immature fruit was initially green and covered with fine prickles at 20 days after flowering (DAF) (Figure 3A). During maturation, the fruit underwent an initial phase of slow growth at 40 DAF, followed by rapid expansion at 60 DAF. Thereafter, the epidermal prickles gradually lignified and hardened at 80 DAF, and the epicarp transitioned from verdant green to golden yellow at full maturity at 100 DAF. Subsequently, we found that flavonoid accumulation increased progressively during fruit maturation as the fruit turned yellow, reaching 5.60 mg/g DW at 80 DAF and ultimately peaking at 6.03 mg/g DW at 100 DAF. In addition, the total flavonoid content in stem was 3.57 mg/g (Figure 3B). Although *RrMYB5* was constitutively expressed throughout fruit development, its transcript abundance increased during the early stages and peaked at the rapid expansion stage of 60 DAF. Thereafter, *RrMYB5* expression declined at 80 and 100 DAF during ripening (Figure 3C). Moreover, *RrMYB5* expression was highest in fruits, reaching 27.66-fold that in leaves, followed by stems and flowers, with transcript levels differing by 15.46- and 3.65-fold relative to leaves, respectively, suggesting that its expression profile was consistent with the pattern of flavonoid accumulation (Figure 3D).

### 2.4. The Indentification of RrMYB5 Overexpression and Expressions of Flavonoid Structrual Genes

**Figure 4:**
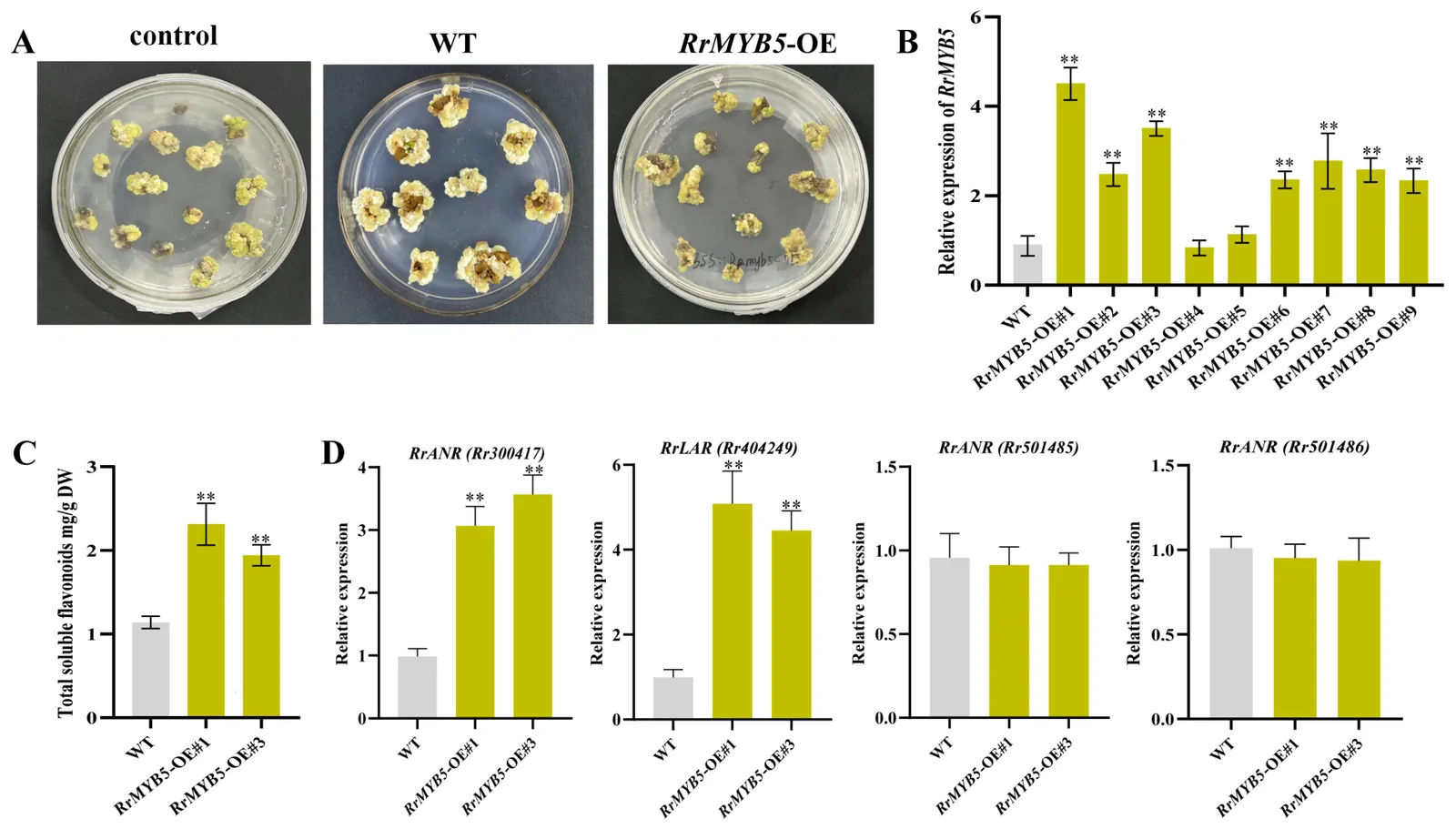
Wild-type and *RrMYB5*-overexpressing *R. roxburghii* calli. (**A**) Phenotypes of negative control and *RrMYB5*-overexpressing calli derived from leaves of *R. roxburghii* after 30 days of culture in 1/2 MS media. (**B**) qRT-qPCR of *RrMYB5* expression in WT and RrMYB5-overexpressed calli. (**C**,**D**) Accumulation of total flavonoids and transcript levels of *RrANRs* and *RrLAT* in WT and overexpressing calli. Data are presented as means ± SD (*n* = 3). ** indicates significant difference at *p* ≤ 0.01, determined by Student’s *t*-test.

To better elucidate the function of *RrMYB5*, it was introduced into the genome of calli derived from *R. roxburghii* leaves via *Agrobacterium*-mediated transformation, producing overexpression transgenic callus lines. PCR analysis with DNA of *R. roxburghii* calli using *vir* gene primers indeed did not display amplification of the *vir* fragment. This was performed to avoid false positives due to persistent Agrobacterium cells on the explant surface. The resulting *RrMYB5*-overexpressing calli exhibited a more compact, pale-yellow, and friable morphology, whereas wild-type (WT) calli developed extensive dark-brown structures (Figure 4A). qRT-PCR analysis confirmed successful overexpression of *RrMYB5*, with *RrMYB5*-OE#1 showing the greatest increases, reaching 5.05- and 3.94-fold higher expression than the WT, respectively (Figure 4B). Soluble flavonoid contents were significantly elevated in RrMYB5-OE calli compared with the WT, reaching 2.33 and 1.96 mg/g, corresponding to 1.05-fold and 71.93% increases over wild-type levels, respectively (Figure 4C). Given that *RrMYB5* is a typical MYBPA-type transcription factor, the expression levels of flavanol pathway genes *RrANR* and *RrLAR* in *RrMYB5*-OE calli were all detected. According to the *R. roxburghii* genome, one *RrLAR* and three *RrANR* transcripts were annotated (Figure 4D). Expression analysis revealed that *RrANR* (*Rr300417*) and *RrLAR* (*Rr404249*) transcript levels were markedly higher than those in the control group, increasing by 3.38- and 4.86-fold, respectively, which was consistent with previous findings [21]. 

### 2.5. The Determination of Flavonoids in RrMYB5-OE Calli by LC-MS Analysis

**Figure 5:**
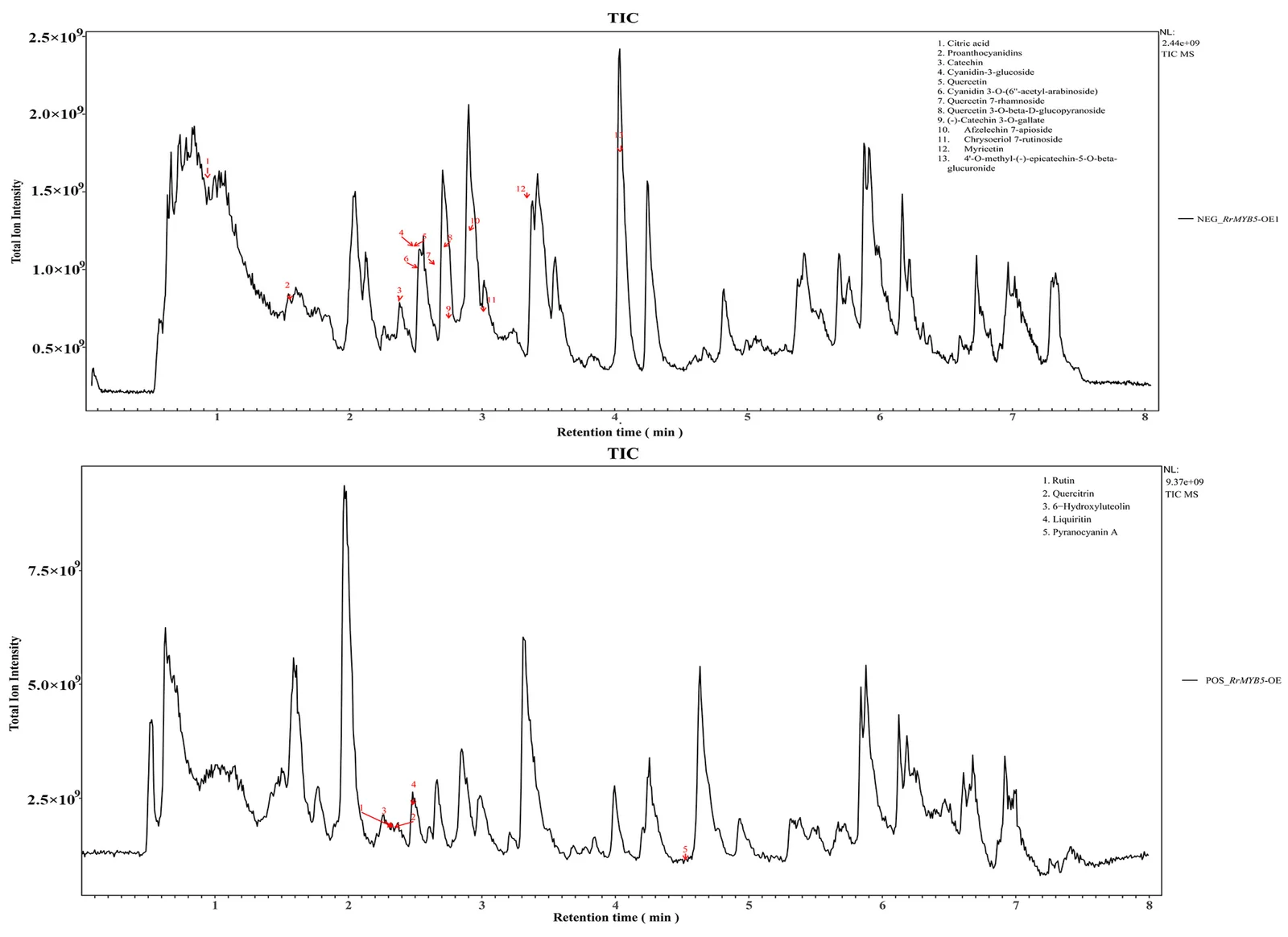
Negative- and positive-ion mass spectra of major bioactive compounds separated by LC-MS. Red arrows indicate the corresponding metabolites. The items labeled 1–13 were defined as follows: citric acid, proanthocyanidins, catechin, cyanidin-3-glucoside, quercetin, cyanidin 3-O-(6″-acetyl-arabinoside), quercetin 7-rhamnoside, quercetin 3-O-beta-D-glucopyranoside, (-)-catechin 3-O-gallate, Afzelechin 7-apioside, chrysoeriol 7-rutinosideand, myricetin and 4′-O-methyl-(-)-epicatechin-5-O-beta-glucuronide. Five compounds were revealed in positive-ion mode, and numbers 1–5 represent rutin, quercitrin, 6-hydroxyluteolin, liquiritin, and proanthocyanidins.

To further define the role of *RrMYB5* in regulating flavonoid accumulation, total ion chromatograms (TICs) were acquired, revealing symmetric peak profiles and consistent metabolite coverage across both ionization modes. Owing to differences in metabolite polarity, compounds in *Rosa roxburghii* fruits were separated by LC-MS and eluted in descending order of polarity. The eluents were subsequently ionized via electrospray ionization, generating characteristic ion response profiles. In negative-ion mode, 13 metabolites were detected. The items labeled 1–13 were defined as follows: citric acid, proanthocyanidins, catechin, cyanidin-3-glucoside, quercetin, cyanidin 3-O-(6″-acetyl-arabinoside), quercetin 7-rhamnoside, quercetin 3-O-beta-D-glucopyranoside, (-)-catechin 3-O-gallate, Afzelechin 7-apioside, chrysoeriol 7-rutinosideand, myricetin and 4′-O-methyl-(-)-epicatechin-5-O-beta-glucuronide. Five compounds were revealed in positive-ion mode, and numbers 1–5 represent rutin, quercitrin, 6-hydroxyluteolin, liquiritin, and proanthocyanidins (Figure 5). The annotations of 18 metabolites were assigned by matching experimental parameters, including retention time (RT), molecular formula, and MS/MS fragment patterns (*m*/*z*) against authentic standards and the reference mass spectral library (Table 1).

Afterwards, the relative abundances of six compounds were semi-quantitatively determined from HPLC chromatograms, with detailed information and their relative abundance provided in Table S2. Box-plot analysis showed that rutin, proanthocyanidins, quercitrin, quercetin and catechin were significantly increased in *RrMYB5*-overexpressing calli, indicating that *RrMYB5* plays a prominent role in modulating the accumulation of flavonols, flavanols, and PAs (Figure S2). By contrast, cyanidin-3-glucoside levels remained unchanged between *RrMYB5*-OE transgenic calli and the wild type.

### 2.6. Genome-Wide Identification of Direct Target Genes of RrMYB5 by DAP-seq

**Figure 6:**
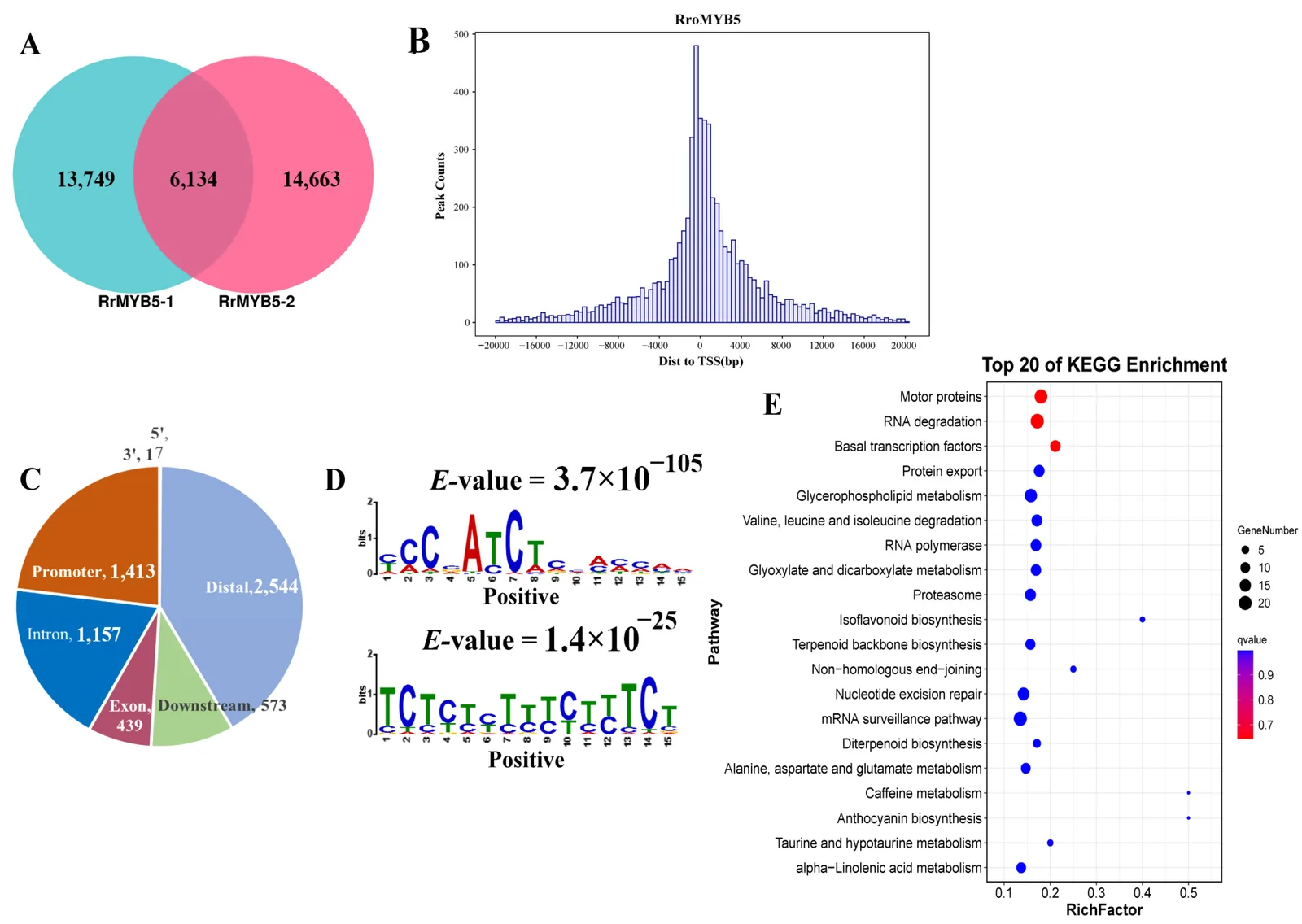
Identification of RrMYB5 target genes by DAP-seq analysis. (**A**) Venn diagram of *RrMYB5* binding peaks revealed by DAP-seq analysis in two replicates. (**B**) Enrichment of *RrMYB5* binding peaks of the transcription start site (TSS). (**C**) Enriched motifs within *RrMYB5* binding peaks. (**D**) The motif shows up as the enriched core sequence. The E value was calculated by MEME (motif-based sequence analysis tool). (**E**) The top 20 KEGG enrichment of genes regulated by RrMYB5.

To investigate the downstream targeted flavonoid genes mediated by *RrMYB5*, we performed DNA affinity purification sequencing (DAP-seq) with two replicates to uncover genome-wide binding events directly targeted by the *RrMYB5* transcription factor. High sequencing quality and data reliability supported subsequent analyses (Table S3). In total, 6134 peaks bound by *RrMYB5* were identified across the two biological replicates using MACS2 with a *q*-value < 0.01 and FC > 2 (Figure 6A). These binding peaks were strongly enriched within 1 kb upstream of the transcription start site (TSS) (Figure 6B). An analysis of their genomic distribution showed a predominant preference for binding to distal regions, with up to 2544 peaks, followed by promoter regions proximal to TSSs, comprising 1413 peaks (Figure 6C). *RrMYB5* also showed preferential binding to introns (1157 peaks), the downstream region (573 peaks), and exons (439 peaks), accounting for 18.86%, 9.34%, and 7.16% of all peaks, respectively (Figure 6B). 

Motif enrichment analysis identified a broad repertoire of related sequences bound by *RrMYB5*, including the conserved [CT]CC[ACG]ATCTC motif (E-value = 3.7 × 10^−105^) and TCTCT[CT]T[CT][CT]CT[CT]TCT as the most highly enriched core motif (E-value = 1.4 × 10^−25^) (Figure 6D). KEGG analysis revealed that the putatively targeted genes were significantly enriched in metabolic pathways associated with flavonoid, flavone, flavonol, and anthocyanin biosynthesis (Figure 6E). Together, these results suggest that *RrMYB5* may coordinate a broad spectrum of biological processes underlying flavonoid metabolism in *R. roxburghii*.

### 2.7. RrMYB5 Promoted the Expression of RrFLS and RrF3H

**Figure 7:**
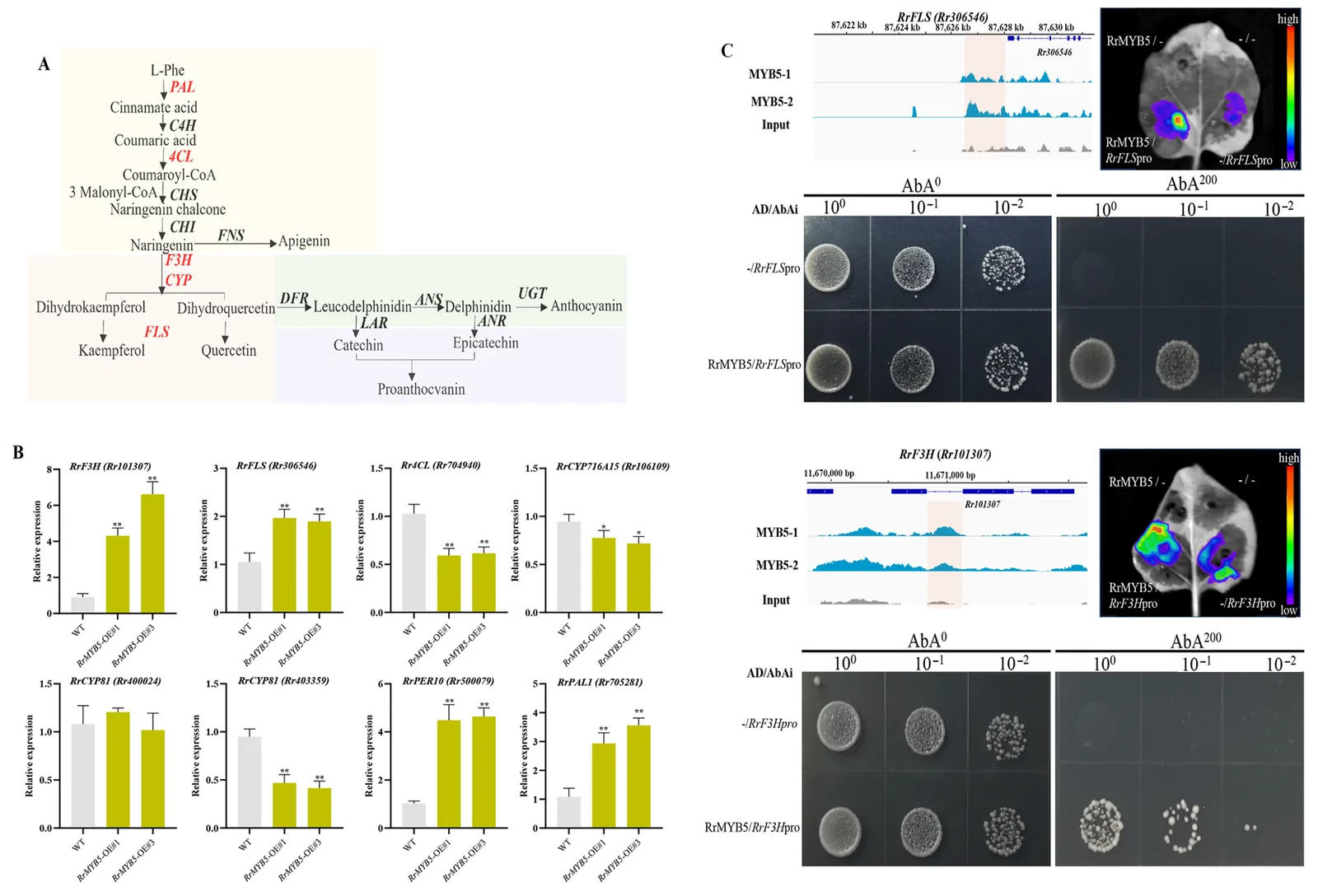
RrMYB5 bound to the promoters of *RrFLS* (*Rr101307*) and *RrF3H* (*Rr306546*) and promoting their expression. (**A**). The proposed flavonoid pathway in *R. roxburghii*, with enzymes putatively regulated by RrMYB5 highlighted in red. (**B**). Transcript levels of eight enzyme-encoding genes. Statistical comparisons were performed using ANOVA followed by Student’s *t*−test, * and ** indicate significant differences *p* ≤ 0.05 and *p* ≤ 0.01, individually. (**C**). Genome browser view of DAP-seq signals at the *RrF3H* and *RrFLS* loci. DAP-seq tracks show RrMYB5 binding peaks and the corresponding input control. RrMYB5 binding to the promoters of *RrF3H* and *RrFLS* was further validated by yeast one-hybrid and dual-luciferase assays.

To elucidate the regulatory genes of *RrMYB5* in flavonoid biosynthesis, candidate genes implicated in this pathway were identified and are highlighted in red, including *PAL*, *4CL*, *F3H*, *CYP*, and *FLS* (Figure 7A). Based on the DAP-seq results, eight genes encoding these enzymes were selected for expression analysis in *RrMYB5*-overexpressing transgenic calli and wild-type controls. The qRT-PCR analysis displayed that five genes were markedly regulated, including *RrF3H* (*Rr101307*), *RrFLS* (*Rr306546*), *RrCYP81* (*Rr403359*), *PER10* (*Rr500079*), and *RrPAL1* (*Rr705281*). Among these, *RrF3H* (*Rr101307*) and *RrFLS* (*Rr306546*) reached average expression levels 6.34- and 1.93-fold higher than those in the WT, respectively (Figure 7B). Given these robust and dynamic changes, the promoters of *RrF3H* and *RrFLS* were selected for further investigation. Integrative Genomics Viewer tracks of DAP-seq data showed increased chromatin accessibility and strong *RrMYB5* binding at the promoter regions of *RrF3H* and *RrFLS* loci (Figure 7C). Yeast one-hybrid assays further confirmed the in vitro interaction of RrMYB5 binding to the promoters of *RrF3H* and *RrFLS*. Moreover, dual-luciferase transient expression assays were conducted in tobacco leaves and indicated that the presence of the *RrMYB5* significantly enhanced LUC activity, corresponding to the transcriptional activation of *RrF3H* and *RrFLS*.

## 3. Discussion

### 3.1. RrMYB5 Is a Positive Regulator of Flavonol or Proanthocyanidin Biosynthesis 

Flavonoids constitute a major class of secondary metabolites in *R. roxburghii* fruit and have diverse bioactivities, including potent antioxidant and anti-inflammatory effects [22,23]. In our study, we determined that the hub *RrMYB5* showed a high correlation with quercetin and catechin, which have been reported as predominant and representative constituents of flavonoids [3]. Conserved-domain analysis further indicated that *RrMYB5* belongs to the typical PA1-specific R2R3-type MYB transcription factor family and shares high similarity with MYB5 from *R. rugosa*, suggesting its potential role in promoting proanthocyanidin accumulation and activating the expression of flavonoid-related genes in *R. roxburghii* [24]. 

*RrMYB5* exhibited high transcript levels in *R. roxburghii* fruits, consistent with the greater accumulation of flavonoids in fruits than in flowers and leaves. Moreover, in relation to the morphological transitions during fruit development, flavonoid accumulation and the expression pattern of *RrMYB5* were investigated across five distinct stages. During the early development stages (20, 40 and 60 DAF), the two profiles showed strong coincidence, whereas *RrMYB5* expressions declined at the later ripening stages. This may contribute to the reduced proanthocyanidin or tannin accumulation, effectively alleviating fruit astringency. However, total soluble flavonoid levels continued to increase during the turning and fully mature stages. It might be due to the progressive accumulation of alternative flavonoid subclasses. For example, anthocyanin accumulation may intensify as fruits reach full maturity, coinciding with the visible transition of the epicarp from green to yellowish or golden coloration, which may redirect metabolic flux toward anthocyanin biosynthesis [25]. In addition, overexpression of PA1-type *MYB* genes has been shown to promote the expression of genes involved in proanthocyanidin accumulation. Consistently, overexpression of *RrMYB5* in *R. roxburghii* calli increased catechin and quercetin contents, and the expression pattern of *RrMYB5* closely resembled those of *RrANR* and *RrLAR*, which encode key enzymes in proanthocyanidin biosynthesis in this study. As proanthocyanidins are potent natural antioxidants, we also observed that *RrMYB5*-overexpressing calli exhibited less tissue browning than wild-type calli, likely owing to enhanced antioxidant capacity. Collectively, these findings indicated that *RrMYB5* acted as a positive regulator of flavonol and proanthocyanidin biosynthesis.

### 3.2. Flavonoid Biosynthetic Genes Could Be Promoted by RrMYB5 in Various Pathways

Most studies have shown that R2R3 MYB interacts with specific bHLH transcription factors and WD40 proteins to assemble ternary MBW complexes that activate target genes. For example, the PA1-type MtMYB5 formed a quaternary complex with MtTT8 and MtWD40, which strongly activated the promoters of MtANR and MtLAR [26]. Likewise, its homolog gene MYB5 in *R. rugosa* could form MBW to activate the expression of LAR and ANR [13]. In our study, overexpression of RrMYB5 resulted in the upregulation of RrLAR and RrANR in *R. roxburghii* calli, potentially through an MBW complex that warrants further verification.

Nevertheless, the N terminus of RrMYB5 comprises two tandem MYB repeats, suggesting that it also constitutes the core domain responsible for specifically recognizing and binding to the downstream target promoter. For example, MYB5 from R. rugosa has been reported to exhibit strong auto-activation activities [13]. In this study, DAP-seq analysis demonstrated that RrMYB5 broadly influences flavonoid biosynthesis and RrMYB5 specifically binds to the promoter regions of the key structural genes RrFLS and RrF3H in the flavonol biosynthetic pathway and activates their transcription [27], resembling the role of JsMYB in regulating flavonol biosynthesis in Juglans sigillata Dode [28]. Taken together, these results indicated that RrMYB5 promoted the expression of flavonoid biosynthetic structural genes through both synergistic and direct regulatory mechanisms, thereby enhancing flavonoid accumulation through multiple regulatory strategies.

### 3.3. RrMYB5 Modulates the Flux of Flavonoid Metabolic Branches Mainly into Flavonols and PA

In addition to the conserved R domain at the N-terminal region, the C-terminus of R2R3 MYB predominantly functions as a transcriptional activation or repression domain. Although PA1-type MYBs commonly regulate proanthocyanidin biosynthesis, their independent evolutionary divergence has generated substantial variation in their C-terminal amino acid sequences [20]. Thus, activation of PA1-type MYBs not only promotes PA accumulation but also redirects precursor metabolic flux away from proanthocyanidin biosynthesis toward flavonol or anthocyanin pathways [12]. This property enables PA1-type MYBs to flexibly modulate flux across distinct branches of flavonoid metabolism [14].

Similarly, previous results showed that total anthocyanin content was significantly reduced in transgenic tobacco overexpressing MYB5 from *R. rugosa*, whereas anthocyanin accumulation increased in transgenic shoots of *R. rugosa*, despite elevated proanthocyanidin levels in both species. These findings indicate that PA1-type MYBs exert species-dependent effects on anthocyanin accumulation [13]. Consistently, our analysis revealed that RrMYB5 might have little positive association with anthocyanin structural genes. Nevertheless, overexpression of *RrMYB5* enhanced PA and quercetin accumulation, implying that RrMYB5 primarily influences structural genes involved in flavanol and flavonol biosynthesis. Collectively, these findings suggested that RrMYB5 might redirect metabolic flux preferentially toward flavonol and proanthocyanidin accumulation rather than anthocyanin biosynthesis [13]. This effect may result from an imbalance in the expression of flavonoid-related genes, such as DFR, ANS, and UFGT, indirectly regulated by RrMYB5. Shared substrates may be competitively allocated among different flavonoid branches, thereby influencing the relative biosynthesis of other flavonoid compounds and anthocyanins. In addition, flavonoid profiling and co-expression network analysis were focused on fruits, flowers and leaves, which were carried out in the roots and stems. Since these organs may exhibit a considerable level of flavonoid accumulation, a more comprehensive regulator might influence expression of these flavonoid structural genes.

## 4. Materials and Methods

### 4.1. Plant Materials and Collection

The seedlings of *R. roxburghii* were cultivated in an open field at Guizhou Normal University in Guiyang, Guizhou, China (26.38° N, 106.63° E). Tissue samples were harvested, encompassing leaves, flowers, and fruits across five distinct developmental phases: at the initial green stage (about 20 days after flowering, 20 DAF) on 20 May 2025, first slow-growth stage 40 DAF on 10 June 2025, rapid expansion stage 60 DAF on 1 July 2025, second slow-growth stage 80 DAF on 21 July 2025, and finally the mature stage 100 DAF on 10 August 2025. All samples were collected between 09:00 and 10:00 a.m. on sunny days to minimize potential variation caused by diurnal changes in environmental conditions and plant physiological status. Some samples were dried at 50 °C and mechanically pulverized into a fine powder, and others were immediately snap-frozen in liquid nitrogen, then stored at −80 °C pending further analysis. 

### 4.2. Determination of Flavonoids and Phenols

A 0.5 g aliquot of each sample was suspended in 5 mL of 80% methanol and extracted by ultrasonic treatment at 55 °C for 1.5 h. This was followed by centrifugation, the supernatant was collected, and the residue was subjected to a second round of ultrasonic extraction and centrifugation. The resulting supernatants were pooled and brought to a final volume of 10 mL for subsequent quantification. Total phenol and flavonoid contents in different tissues and fruit developmental stages of *R. roxburghii* were quantified by G0117W and G0118W (Suzhou Grace Biological Company, Suzhou, China) assay kits. All analyses were performed with three biological replicates according to the manufacturer’s protocol [29]. A sample solution was filtered through 0.45 μm organic membranes. Chromatographic separation was achieved on a C18 column (4.6 × 150 mm, 5 μm). The ultraviolet (UV) detection wavelength was set at 280 nm, with the column temperature maintained at room temperature. The system operated at a constant flow rate of 1 mL/min with an injection volume of 20 μL. The mobile phase consisted of Eluent A (0.8% acetic acid in water) and Eluent B (acetonitrile) as follows: 0–10 min, 5–10% B; 10–20 min, 15% B; 20–30 min, 25–30% B; 30–40 min, 30–40% B; 40–50 min, 40–50% B; 50–55 min, 50–100% B; 55–60 min, 5% B.

### 4.3. WGCNA Analysis and Construction of Co-Expression Network 

Functional unigenes were annotated by BLAST (https://blast.ncbi.nlm.nih.gov/Blast.cgi (accessed on 5 August 2026)). The raw RNA-Seq data of leaves, flowers, and fruits are publicly available in the NCBI GEO database under the accession number GSE122014. DEGs among flowers, leaves, and fruits of *R. roxburghii* have been identified as previously described [19]. Highly co-expressed gene modules associated with flavonoid accumulation were constructed using FPKM values of DEGs and the WGCNA package in R software [16]. The WGCNA network and module were generated using an unsigned topological overlap matrix (TOM). The kME (for modular membership, also known as eigengene-based connectivity) was determined as the Pearson correlation coefficient between each gene and the corresponding module eigengene to assess the association between modules and total flavonoid content. A correlation network was constructed based on the most significant module of genes with the threshold being a Pearson correlation coefficient within the module greater than 0.8 and a *p*-value less than 0.05, and visualized using Cytoscape (v3.7.2, https://cytoscape.org/).

### 4.4. Sequence Analysis and Phylogenetic Tree

Total RNA extracts were used as templates, together with gene-specific primers (Table S1), to amplify the coding sequence (CDS) of *RrMYB5*. Conserved domains were predicted using SMART (https://smart.embl.de/ (accessed on 5 August 2026)) and ESPript 3.0 (https://espript.ibcp.fr/ESPript/ESPript/ (accessed on 5 August 2026)) websites. For phylogenetic tree analysis, multiple sequence alignments were performed through the Clustal W program with default parameters (https://www.genome.jp/tools-bin/clustalw) (accessed on 5 August 2026). The resulting alignments were submitted to MEGA 10 (https://www.megasoftware.net/older_versions) (accessed on 5 August 2026) to construct an unrooted phylogenetic tree using a neighbor-joining statistical method, with branch support assessed by bootstrap analysis based on 1000 replicates.

### 4.5. Subcellular Location

The CDS of RrMYB5 was amplified and inserted into the *Xba*I-digested pM999-eGFP vector, which carries an enhanced green fluorescent protein (eGFP) tag driven by the CaMV 35S promoter. The recombinant and empty-vector plasmids were independently transformed into *Arabidopsis* suspension protoplasts via PEG (polyethylene glycol) transformation [30]. Fusion protein localization was examined 24 h after transformation. Fluorescence signals were visualized using a laser scanning confocal microscope (SP8, Leica, Wetzlar, Germany) at an excitation wavelength of 488 nm and an emission wavelength of 509 nm.

### 4.6. RNA Extraction and Quantitative RT-PCR

RNA was extracted from fruits or different tissues using the Trizol method (Trizol, Takara Bio, Kusatsu, Shiga, Japan) with the addition of RNAiso-mate to remove polysaccharides and polyphenol molecules. Then RNA was reverse-transcribed using RT-PCR Kit (TaKaRa, Maebashi, Japan) with an oligo dT-adaptor primer. Quantitative real-time RT-PCR was performed using SYBR Premix Ex Taq (TaKaRa). Amplification was carried out under the following cycling parameters: denaturation for 10 min at 95 °C, followed by 40 cycles of denaturation at 95 °C for 15 s, annealing at 55 °C for 30 s, and extension at 72 °C for 30 s. Primers were designed using Primer Premier 6 and are listed in Table S1. *Rractin* and *ubiquitin (RrUBQ)* were used as two internal controls, and relative gene expression levels were calculated using the 2^−ΔΔCt^ method [19,31]. Three biological replicates and three technical replicates were performed to ensure the reproducibility of the results.

### 4.7. Vector Construction and Transgenic Generation of R. roxburghii Calli

The CDS of *RrMYB5* was cloned into pCAMBIA1300 via the *Kpn*I and *Bam*HI restriction sites to generate an overexpression vector. The resulting constructed vector was subsequently transformed into *A. tumefaciens* strain GV3101. For *R. roxburghii* transformation, calli were induced from leaves on 1/2 MS medium supplemented with 2% sucrose, 0.3 mg/L indole-3-butyric acid (IBA), 0.7% agar, pH 5.8, for 30 days in the dark. The calli were then incubated with the *Agrobacterium* GV3101 respectively harboring the empty vector (pCAMBIA1300 alone) which was used as a control and the constructed construct in co-cultivation buffer containing half-strength MS medium, 2% sucrose, and 0.4 mg/mL acetosyringone for 1 h in the dark with gentle shaking. The infected calli were maintained on co-cultivation medium for 3 days in the dark, then washed with sterile water and put onto the 1/2 MS medium with antibiotics (250 mg/L carbenicillin, 250 mg/L timentin, and 4 mg/L hygromycin) for one week and then moved to fresh medium. Following antibiotic selection and RT-PCR confirmation (Table S1), positive overexpression calli were selected for further analysis.

### 4.8. LC-MS Metabolomic Analysis

Approximately 50 mg of calli was weighed and transferred into a 2 mL centrifuge tube containing a 6 mm grinding bead. Extraction was performed using 400 μL of ice-cold methanol:water (4:1, *v*/*v*) supplemented with L-2-chlorophenylalanine (0.02 mg/mL) as internal standards. Samples were homogenized using a cryogenic grinder at −10 °C, and 50 Hz for 6 min, followed by ultrasonication at 5 °C and 40 kHz for 30 min. After incubation at −20 °C for 30 min, the samples were centrifuged at 13,000× *g* for 15 min at 4 °C, and the supernatants were collected and filtered through 0.45 μm organic membranes. Chromatographic separation was achieved on a C18 column (ACQUITY, 100 × 2.1 mm, 1.7 μm). The ultraviolet (UV) detection wavelength was set to 280 nm, with the column temperature maintained at 40 °C. The system was operated at a constant flow rate of 1 mL/min with an injection volume of 2 μL. The mobile phase consisted of Eluent A (2% acetonitrile in water and 0.1% formic acid) and Eluent B (acetonitrile containing 0.1% formic acid). Electrospray ionization (ESI) was performed in both positive- and negative-ion modes at the *m*/*z* scan range of 70 to 1050, with resolutions of 60,000 (MS^1^) and 7500 (MS^2^). Spray voltage: +3500 V (positive), −3000 V (negative). Quality-control (QC) samples were injected after every 5–15 injections to monitor instrument stability [32]. Raw data were processed using Progenesis QI v3.0 (Waters, Milford, MA, USA), and analytical stability was assessed by z-score analysis [33]. Features were extracted, aligned, and filtered according to retention time, *m*/*z*, and peak intensity against the Majorbio Plant Metabolite Database (MJDBPM) and the Human Metabolome Database (HMDB). Metabolites were semi-quantified based on peak areas. All analyses were performed with three biological replicates according to the established protocols [34].

### 4.9. Library Construction and DAP Sequencing

The CDS of *RrMYB5* was amplified and subsequently cloned into the pHalo-Tag for prokaryotic expression of recombinant RrMYB5. Genomic DNA from *R. roxburghii* was extracted, and enriched DNA was sheared into short fragments by ultrasonication. The fragmented DNA was then end-repaired, 3′ A-tailed, and ligated to Illumina sequencing adapters. DNA fragments of appropriate size, typically 100–300 bp including adapter sequences, were selected and PCR-amplified. Qualified libraries were subsequently sequenced on an Illumina NovaSeq 6000 platform by Gene Denovo Biotechnology Co. (Guangzhou, China). After raw-read filtering, clean reads from each sample were aligned to the reference genome with accession number CNP0004212 (https://db.cngb.org/cnsa/) (accessed on 5 August 2026) using Bowtie2 v2.2.5 [35,36]. Reads mapped across gene bodies, from transcription start sites (TSSs) to transcription termination sites (TESs), as well as 2 kb upstream and downstream flanking regions, were quantified using deepTools [37]. MACS2 4 was designed to identify read-enriched regions from DAP seq data [38]. A dynamic Poisson distribution was applied to calculate the *p*-value for a specific region based on unique mapped reads, and regions with q-values < 0.05 were defined as peaks. The resulting *p* values were adjusted using false discovery rate (FDR) correction, with FDR < 0.05 set as the significance threshold. Pathways meeting this criterion were considered significantly enriched among peak-associated genes [39].

### 4.10. Vector Construction and Yeast One-Hybrid Assay

We amplified the promoter sequences of *RrFLS* (*Rr306546*) and *RrF3H (Rr101307*) from *R. roxburghii* genomic DNA and cloned them into the bait pAbAi vector (Clontech, San Jose, CA, USA). The recombinant bait plasmids were linearized and subsequently transformed into Y1HGold cells. To generate the pGADT7-*RrMYB5* effector plasmid, the CDS of *RrMYB5* was amplified using primers (Table S1), digested with *Eco*RI and *Bam*HI, and subsequently ligated into the connected to pGADT7 vectors. Transformants were isolated on synthetic dextrose (SD) medium. After determining the minimal inhibitory concentration of aureobasidin A (AbA), the effector plasmid was introduced into yeast cells and selected using SD/-Leu supplemented with AbA. Yeast cells were cultured in SD-Leu/AbA medium for 2~3 days.

### 4.11. Dual Luciferase Complementation Assay

The *RrMYB5* CDS was inserted into the reporter vector pGreenII 62-SK. Meanwhile, the promoter sequences of *RrFLS* (*Rr306546*) and *RrF3H* (*Rr101307*) were amplified from *R. roxburghii* genomic DNA and individually cloned into the effector vector pGreenII 0800-LUC plasmid. The resulting constructs were introduced into *Agrobacterium tumefaciens* strain GV3101 carrying the pSoup plasmid. Each effector–reporter combination was co-infiltrated into *N. benthamiana* leaves. All primers are listed in Table S1, and each experiment was performed with a minimum of three biological replicates. 

### 4.12. Statistical Analysis

Three independent biological replicates were included in this study. All data are expressed as mean ± standard deviation (SD), and statistical significances were analyzed by a one-way analysis of variance (ANOVA) followed by Student’s *t*-test using Statistical Product and Service Solutions version 20.0 (SPSS Inc., Chicago, IL, USA). In addition, one-way ANOVA followed by Duncan’s multiple range test was applied for multiple comparisons.

## Figures and Tables

**Table 1:** Identification of the main phytochemical compounds in calli of *R. roxburghii* by LC-MS analysis.

Metabolite	Mode	Class	RT (min)	*m*/*z*
Pyranocyanin A	Positive	Pyrano-anthocyanin	4.5253	675.2128
Rutin	Positive	Flavonols, O-glycoside	2.3388	611.1612
Quercitrin	Positive	Flavonols, O-glycoside	2.3388	449.1078
6-Hydroxyluteolin	Positive	Flavones	2.3388	303.0498
Quercetin 7-rhamnoside	Negative	Flavonols, O-glycoside	2.6419	447.0934
Quercetin 3-O-beta-D-glucopyranoside	Negative	Flavonols, O-glycoside	2.7096	463.0883
Chrysoeriol 7-rutinoside	Negative	Flavones	3.0093	607.1664
Afzelechin 7-apioside	Negative	Flavanol	2.9058	857.2491
Cyanidin 3-O-(6″-acetyl-arabinoside)	Negative	Anthocyanins	2.517	496.078
Citric acid	Negative	Organic acids	0.9449	191.0194
Catechin	Negative	Flavanol	2.33	289.0722
4′-O-methyl-(-)-epicatechin-5-O-beta-glucuronide	Negative	Flavanol	3.981	459.1295
(-)-Catechin 3-O-gallate	Negative	Flavanol	2.7479	441.0828
Cyanidin-3-glucoside	Negative	Anthocyanins	2.4804	430.09
Liquiritin	Positive	Flavanone	2.4781	473.1444
Myricetin	Negative	Flavonols	3.3376	681.0741
Quercetin	Negative	Flavonols	2.4804	283.0242
Proanthocyanidins	Negative	Proanthocyanidin	1.5521	591.1559

## Data Availability

The DAP-seq raw sequencing data generated in this study have been deposited in the NCBI Sequence Read Archive (SRA) under BioProject PRJNA1473525.

## Supplementary Materials

The following supporting information can be downloaded at: https://www.mdpi.com/article/10.3390/plants15162428/s1, Figure S1. The components and contents of different flavonoids in leaf, petal and fruit of *R. roxburghii*. Data are presented as means ± SD (n = 3); Figure S2. Comparative analysis of flavonoid levels in RrMYB5-overexpressing transgenic calli and wild-type controls. RrMYB5-OE1/2/3 (black, red, and blue) and WT1/2/3 (purple, yellow, and green). Arrows indicate the retention time corresponding to the peaks of each compound. The y-axis represents absolute signal intensity. Red and blue boxes represent the RrMYB5-OE and WT groups, respectively. Statistical comparisons were performed using ANOVA followed by Student’s *t*-test; * indicates significant differences *p* ≤ 0.05; Table S1. List of the primers used in this study; Table S2. The expression of compounds in calli of *R. roxburghii* by LC-MS analysis; Table S3. The data for quality control of DAP-seq.
